# Editorial: Efficient AI in particle physics and astrophysics

**DOI:** 10.3389/frai.2022.999173

**Published:** 2022-09-30

**Authors:** Javier Duarte, Mia Liu, Jennifer Ngadiuba, Elena Cuoco, Jesse Thaler

**Affiliations:** ^1^Department of Physics, University of California, San Diego, La Jolla, CA, United States; ^2^Department of Physics, Purdue University, West Lafayette, IN, United States; ^3^Particle Physics Division, Fermi National Accelerator Laboratory, Batavia, IL, United States; ^4^European Gravitational Observatory, Cascina, Italy; ^5^Scuola Normale Superiore, Pisa, Italy; ^6^Center for Theoretical Physics, Massachusetts Institute of Technology, Cambridge, MA, United States; ^7^The NSF AI Institute for Artificial Intelligence and Fundamental Interactions, Cambridge, MA, United States

**Keywords:** efficiency, FPGA, physics-inspired, neural network, high energy physics, particle astrophysics, pruning, quantization

Efficient artificial intelligence (AI) (Sze et al., 2020) is increasingly essential for high energy physics and particle astrophysics applications, especially multi-messenger astronomy, often in the context of accelerated AI for real-time, low-latency, low-memory, or low-power system requirements. AI efficiency may be quantified in different ways depending on the context: from fewer model parameters to fewer operations or greater speed during training or inference. Methods for improving efficiency include, but are not limited to, utilization of specialized hardware, custom neural network structures, pruning parameters, quantization of parameters, efficiency-aware training, knowledge distillation, physics-inspired models, and embedded symmetries or equivariance. Within this scope, this Research Topic collects six papers featuring reviews, case studies, applications, and new approaches exploring efficient AI in high energy physics and particle astrophysics, including computational, data, and conceptual efficiency.

In “*Ps and Qs: Quantization-aware pruning for efficient low latency neural network inference*,” Hawks et al. study machine learning (ML) implementations optimized for inference in hardware *via* pruning, removing insignificant synapses, and quantization, reducing the precision of the calculations. Specifically, the work explores the interplay between pruning and quantization during the training of neural networks for ultra low latency applications targeting high energy physics use cases. By studying various configurations of quantization-aware pruning, the authors find more computationally efficient models than pruning or quantization alone, and Bayesian hyperparameter optimization. They also study the effect of these approaches on neural efficiency (Schaub and Hotaling, 2020) and generalizability.

In “*Inference-optimized AI and high performance computing for gravitational wave detection at scale*,” Chaturvedi et al. develop a new approach in the application of AI to gravitational wave detection where NVIDIA TensorRT is leveraged to optimize the computing time needed for the inference of an ensemble of AI models. The authors developed the software needed to optimally distribute inference over 20 nodes in the ThetaGPU supercomputer at Argonne Leadership Computing Facility—the equivalent of 160 NVIDIA A100 Tensor Core GPUs. With this setup, the authors could process an entire month of the Advanced Laser Interferometer Gravitational-Wave Observatory (aLIGO) data within 50 s, i.e., 3 times faster compared to traditional AI models, while retaining full sensitivity in identifying binary black hole mergers.

In “*Nonlinear noise cleaning in gravitational-wave detectors with convolutional neural networks*,” Yu and Adhikari study nonlinear noise mitigation using convolutional neural networks (CNNs) for gravitational-wave (GW) detection experiments. Currently, the sub-60 Hz sensitivity of GW detectors like aLIGO is limited by the control noises from auxiliary degrees of freedom which nonlinearly couple to the main GW readout. The authors adopt an explicit “slow × fast” structure in the CNN design to handle the bilinear noise coupling that can be viewed as fast channels modulated by slow channels and achieve a factor of a few noise reduction in both GW main readout and auxiliary sensors. It was further demonstrated that the CNN performs well with curriculum learning techniques by combining data from quiet times and periods with active noise injections.

In “*Graph neural networks for charged particle tracking on FPGAs*,” Elabd et al. develop field-programmable gate array (FPGA) implementations of graph neural network (GNN) algorithms for charged particle tracking at the CERN LHC. The authors introduce an automated translation workflow, integrated into hls4ml (Duarte et al., 2018), for converting GNNs into FPGA firmware. Using the public TrackML challenge dataset (Amrouche et al., 2020), they benchmark GNN designs targeting different graph sizes, task complexites, and latency/throughput requirements. One implementation is optimized for low-latency (less than 4 μs) and high-throughput (2.22 MHz or greater) typical for applications in the FPGA-based level-1 trigger systems at the LHC (CMS Collaboration, 2020), while another is optimized to minimize the FPGA resources needed and scales to larger graph sizes (thousands of nodes and edges).

In “*Applications and techniques for fast machine learning in science*,” Deiana et al. review the work being carried out by the scientific community to integrate AI methods into the real-time experimental data processing loop to accelerate scientific discovery. By summarizing two workshops held by the Fast ML for Science community, this report includes the description of a variety of scientific domains including existing work and applications for embedded AI; potential overlaps across scientific domains in data representation or system constraints; and an overview of state-of-the-art techniques for efficient ML and compute platforms, both cutting-edge and speculative technologies.

Finally, in “*Real-time inference with 2D convolutional neural networks on field programmable gate arrays for high-rate particle imaging detectors*,” Jwa et al. develop a custom implementation of a 2D CNN on a Xilinx UltraScale+ FPGA as a viable application for real-time data selection in high-resolution and high-rate particle imaging detectors. To meet FPGA resource constraints, the authors optimize the accuracy and latency of a two-layer CNN with KerasTuner and further optimize the network quantization to minimize the computing resource utilization. The authors use an automated translation workflow for CNN supported in hls4ml (Duarte et al., 2018) tools and achieve the first-ever exploration of employing 2D CNNs on FPGAs for the future Deep Underground Neutrino Experiment (DUNE).

## Author contributions

All authors listed have made a substantial, direct, and intellectual contribution to the work and approved it for publication.

## Funding

JD is supported by the U.S. Department of Energy (DOE), Office of Science, Office of High Energy Physics Early Career Research program under Award No. DE-SC0021187, and by the National Science Foundation (NSF) under Cooperative Agreement OAC-2117997 (The NSF HDR Instiutte for Accelerated AI Algorithms for Data-Driven Discovery, https://a3d3.ai). JN is supported by Fermi Research Alliance, LLC under Contract No. DE-AC02-07CH11359 with the DOE, Office of Science, Office of High Energy Physics. JT is supported by the DOE Office of High Energy Physics under Grant No. DE-SC0012567, and by the NSF under Cooperative Agreement PHY-2019786 (The NSF AI Institute for Artificial Intelligence and Fundamental Interactions, https://iaifi.org).

## Conflict of interest

The authors declare that the research was conducted in the absence of any commercial or financial relationships that could be construed as a potential conflict of interest.

## Publisher's note

All claims expressed in this article are solely those of the authors and do not necessarily represent those of their affiliated organizations, or those of the publisher, the editors and the reviewers. Any product that may be evaluated in this article, or claim that may be made by its manufacturer, is not guaranteed or endorsed by the publisher.
